# You Turn Me Cold: Evidence for Temperature Contagion

**DOI:** 10.1371/journal.pone.0116126

**Published:** 2014-12-31

**Authors:** Ella A. Cooper, John Garlick, Eric Featherstone, Valerie Voon, Tania Singer, Hugo D. Critchley, Neil A. Harrison

**Affiliations:** 1 Brighton and Sussex Medical School, University of Sussex, Brighton, United Kingdom; 2 Institute of Cognitive Neuroscience, UCL, London, United Kingdom; 3 Wellcome Trust Centre for Neuroimaging, UCL, London, United Kingdom; 4 Behavioural and Clinical Neurosciences Institute, University of Cambridge, Cambridge, United Kingdom; 5 Department of Social Neuroscience, Max Planck Institute for Human Cognitive and Brain Sciences, Leipzig, Germany; 6 Sackler Centre for Consciousness Science, University of Sussex, Brighton, United Kingdom; 7 Sussex Partnership NHS Trust, Brighton, United Kingdom; The University of Queensland, Australia

## Abstract

**Introduction:**

During social interactions, our own physiological responses influence those of others. Synchronization of physiological (and behavioural) responses can facilitate emotional understanding and group coherence through inter-subjectivity. Here we investigate if observing cues indicating a change in another's body temperature results in a corresponding temperature change in the observer.

**Methods:**

Thirty-six healthy participants (age; 22.9±3.1 yrs) each observed, then rated, eight purpose-made videos (3 min duration) that depicted actors with either their right or left hand in visibly warm (warm videos) or cold water (cold videos). Four control videos with the actors' hand in front of the water were also shown. Temperature of participant observers' right and left hands was concurrently measured using a thermistor within a Wheatstone bridge with a theoretical temperature sensitivity of <0.0001°C. Temperature data were analysed in a repeated measures ANOVA (temperature × actor's hand × observer's hand).

**Results:**

Participants rated the videos showing hands immersed in cold water as being significantly cooler than hands immersed in warm water, F_(1,34)_ = 256.67, p<0.001. Participants' own hands also showed a significant temperature-dependent effect: hands were significantly colder when observing cold vs. warm videos F_(1,34)_ = 13.83, p = 0.001 with post-hoc t-test demonstrating a significant reduction in participants' own left (t_(35)_ = −3.54, p = 0.001) and right (t_(35)_ = −2.33, p = 0.026) hand temperature during observation of cold videos but no change to warm videos (p>0.1). There was however no evidence of left-right mirroring of these temperature effects p>0.1). Sensitivity to temperature contagion was also predicted by inter-individual differences in self-report empathy.

**Conclusions:**

We illustrate physiological contagion of temperature in healthy individuals, suggesting that empathetic understanding for primary low-level physiological challenges (as well as more complex emotions) are grounded in somatic simulation.

## Introduction

Adaptive social behavior is dependent on the efficient communication of affective and motivational signals between individuals that together facilitate understanding of others' mental and emotional states. In humans, perception of these signals is associated with a marked tendency to mimic, which is well described for emotional facial expressions [1], body postures [2], gesticulations [3] and elements of speech [4]. This tendency, which typically occurs without conscious intent, has been proposed to facilitate emotional understanding across individuals. Empirical data demonstrating correlations between tendency to mimic emotional facial expressions and self-report measures of empathy [5], [6] support the encapsulation of mimicry within the broader concept of empathy [7].

Following de Vignemont and Singer we define empathy as occurring if: ‘(i) one is in an affective state; (ii) this state is isomorphic to another person's affective state; (iii) this state is elicited by the observation or imagination of another person's affective state; (iv) one knows that the other person is the source of one's own affective state' [8]. In contrast we define the narrower concept of emotional contagion as sharing of affect (points i, ii and iii above) in the absence of an awareness that the other is the source of one's own affective state (point iv above).

Though somatically mediated motor signals have traditionally dominated research in this field, recent evidence has demonstrated that effects of emotion contagion can be observed for facial temperature [52] and have even been observed at the level of hormones [9]. Thus, observing a familiar person or a stranger undergoing a Trier Stress test increases cortisol levels not only in the stressed person but also passive observers watching the scene through a one-way mirror or TV screen. Similarly, evidence for contagion effects have also been observed at the level of the autonomic nervous system in the domain of facial flushing, pupil size and skin temperature. For example, pupillary signals have demonstrated a role in signalling the intensity of sadness [6], [10] and skin temperature the experience of anger [11], [12]. Perceptual sensitivity to another's pupil size during sadness has also been shown to predict inter-individual differences in empathy [6]. Similar to somatic motor responses, autonomic contagion has also been described during social exchange. For example, during psychotherapy heart rates of therapists and their clients tend to speed up and slow down together [13]. Pupil size has also been demonstrated to decrease during both the experience [14] and observation of sadness in others [10]. However, whether such contagious effects generalize across different axes of the autonomic nervous system is currently unknown.

Until a decade ago, neuroscientific approaches to the study of empathy were lacking. However, with the discovery of mirror neurons within the premotor cortex, which respond during both performance and observation of a conspecific performing the same action, a potential neural mechanism mediating how we understand other people's actions and intentions was proposed [15], [16]. Shortly after the formulation of such action–perception models of motor behavior to imitation [17], they were extended to the domain of emotions with the first empathy models [18] suggested that perception of another's emotional state should automatically activate a similar representation within the viewer together with associated autonomic and somatic responses.

Subsequently, a huge number of human functional imaging studies have provided empirical support for such shared networks in the domain of feeling and emotional states. Most have been performed in the domain of pain and show an overlapping anterior insula and anterior midcingulate cortex (aMCC) network underlying both the first-hand experience of pain as well as its observation in others [19], [20] for meta-analyses see [21], [22]. Such shared neuronal networks of empathy have also been observed in the domain of neutral touch [23]–[25] as well as pleasant and unpleasant touch [26], [27] disgust and taste [28], [29] as well as positive affect such as joy or reward [30], [31]. In sum, multiple studies have found evidence for our human capacity to share affective states with each other, be it at the level of motor mimicry, autonomic or the neuronal activity.

To date however, no study has asked whether sharing of autonomic physiological responses also extends to peripheral skin temperature. All homeothermic animals including humans rigidly regulate their core body temperature through a variety of involuntary thermoregulatory responses, such as shivering and non-shivering thermogenesis, cutaneous vasomotor responses, sweating, piloerection and panting [32]. Of these, sympathetically mediated changes in peripheral skin blood-flow (manifest as a change in peripheral temperature), is the most acutely sensitive to environmental temperature change [33]. However, changes in peripheral body temperature are additionally linked to changes in emotional state [11], [12], e.g. hot, clammy hands in anxiety or facial flushing in embarrassment, and can be modulated by mental imagery, hypnotic suggestion [34] and disruption of the sense of body ownership using the rubber hand illusion [35] and illusory self-identification with an avatar [64]. Together these findings suggest sensitivity of peripheral body temperature to top-down cognitive processes and a complimentary role in social communication.

To investigate emotional contagion in the domain of body temperature we measured the left and right hand temperature of thirty-six healthy volunteers while viewing videos of two actors placing their right or left hand in warm or cold water. We predicted: 1) that viewing another's hand in warm/cold water in the absence of any emotional cues would be associated with congruent temperature changes in the viewer's hand. 2) That viewed changes in the right hand would be associated with congruent (contagious) temperature changes in the viewer's left hand (reflectional symmetry). 3) That these temperature changes would occur in the absence of more general measures of arousal e.g. change in heart rate. 4) That contagion of another's peripheral temperature change would be greater in participants with high emotional empathy as measured through psychological trait questionnaires.

## Methods

### Participants

Thirty-eight healthy participants with normal or corrected to normal vision were recruited via advertisement on the UCL psychology online research website. Two participants were subsequently excluded from the analysis of temperature responses due to technical failure and 14 from the heart rate analysis due to battery failure. Thus a total of 36 participants (13 males, mean 22.9±3.1 years) were included in analysis of temperature response and 22 (8 males, mean 22.9±3.5 years) in the combined analyses of heart rate and temperature responses. Written informed consent was obtained in accordance with the declaration of Helsinki (Helsinki 1991) and the procedures were approved by the joint Ethics Committee of the National Hospital and Institute of Neurology, London.

### Video temperature stimuli

**Figure 1:**
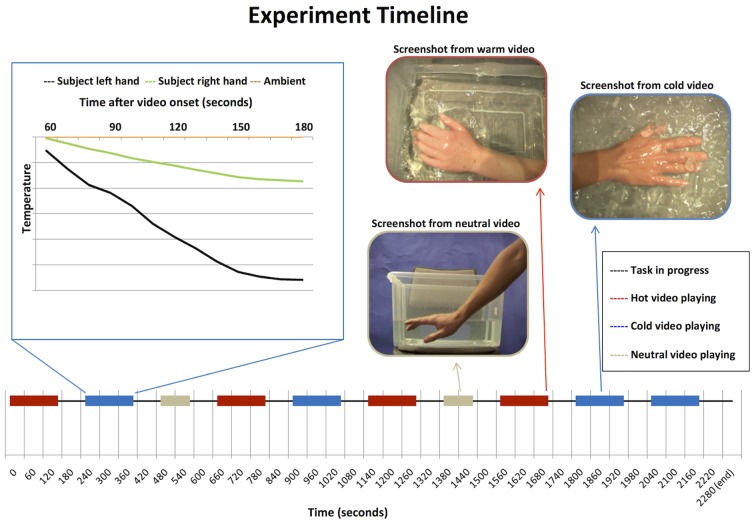
Experimental Timeline. The experimental timeline along the bottom shows video playback, red bars represent playback of warm, blue cold and beige control videos. The graph on the top left shows an example of single participants left (black) and right (green) hand temperature responses to a single illustrative cold video. The orange line illustrates changes in ambient temperature. The pictures on the top right show snap shots from the warm, cold and neutral videos.

Ten custom temperature stimuli videos were produced. Each video began with one of two actors (one male, one female) sitting in front of a transparent container partially filled with water. In four of the videos the actor then gradually added hot water from a steaming kettle into the container, checking the temperature of the water every few seconds with one hand. One video showed the male actor placing his right hand in the water and one his left. The other two videos showed the same procedure with the female actor. Four additional videos showed each actor filling the container with a bag of ice then testing the cold water with his/her left or right hand. The first 40 s of each video showed the actor cautiously filling the semi-filled transparent container with water from a steaming kettle or ice from a bag and intermittently testing the water temperature with his or her hand. The remainder of the videos used in subsequent analyses focused exclusively on the actor's hand placed in the water. These sections of the videos showed the water and the actor's hand only with no facial or other body movement cues that may communicate emotional state. Two additional control videos (each of 120 s duration) showed the same combination of factors i.e. bowl of water and actors hand, however in these videos no hot water/ice was added to the water and the actor's hand was held in front of the water container. One control video showed the female actor's left hand and the other the male actors' right hand (screen-shots illustrated in Fig. 1).

### Study design

The study adopted a randomised within-subject design, with each participant viewing all ten of the videos in counter-balanced order. After each video participants gave subjective ratings for both the observed water temperature (“How Hot or Cold is the Water?”) and the temperature of the actor's hand (“How Hot or Cold is the Actor's Hand?”) using a keyboard controlled visual analogue scale ranging from ‘Very Cold’ (far left) through ‘Neutral’ (centre) to ‘Very Hot’ (far right). Each video was separated by a 60 second inter-trial interval. The task was written and presented, and behavioural responses logged via a desktop computer running Cogent software on a Matlab platform (Mathwork, Nantick MA). See Fig. 1 for study timeline.

### Physiological data recording

All testing was performed in a dedicated testing room kept at a constant temperature of 21°C. Participant's right and left hand temperature and heart rate, as well as ambient room temperature, were recorded throughout the study. Minute (3×2 mm) thermistors were attached to the palmar distal phalanges on the fourth finger of each hand to measure temperature change. Convection and conductive heat transfer to the environment was minimised by attaching the thermistors with micropore tape and placing participant's hands palm upwards on cushions throughout video playback. Ambient temperature was recorded throughout using a third thermistor suspended 20–30 cm in front of the hands. To minimise muscle movement related temperature change participants were asked to keep their hands as still as possible during video payback and use the index finger of both hands to input responses once the video had finished playing. Heart rate was simultaneously monitored using a pulse oxymeter (Nonin 8600; Nonin Medical) attached to each participant's left small finger.

**Figure 2:**
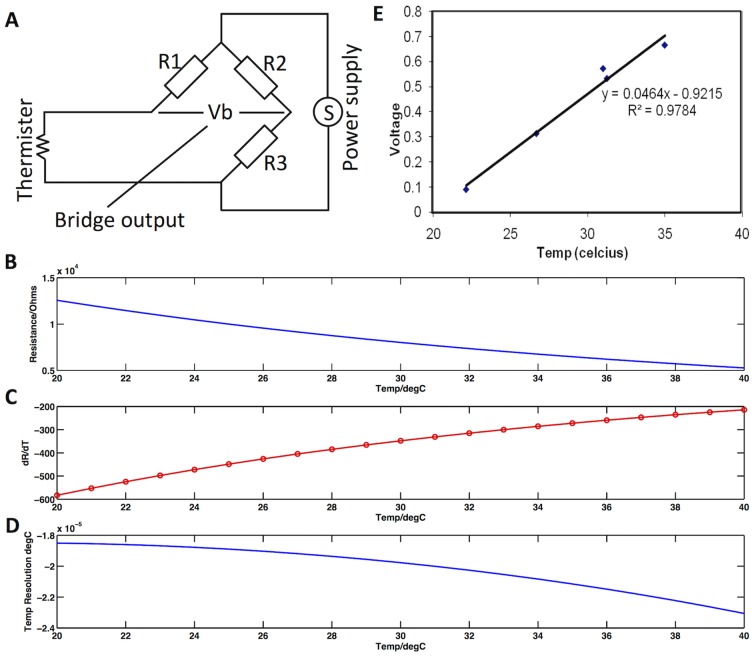
Experimental Setup. Graphic illustrating the experimental setup. Thermistors were attached to the participants' left and right hands with a third thermistor used to record ambient temperature. All three thermistors were connected to Wheatstone bridges (labelled Thermistor transducer) with the output passed to a CED 1902 signal amplifier. Output from the CED 1902 and the Pulse oxymeter (attached to the left hand) were fed into a CED Power 1401 data acquisition interface and the digitized output recorded in a PC running Spike2. A second PC running cogent in Matlab presented all of the task stimuli and passed a timing pulse to the CED Power 1401 to ensure accurate temporal alignment of the data.

Each thermistor was connected to a separate Wheatstone bridge (detailed below) with outputs, pulse oximetry signals and stimulus timing pulses all passed to a Cambridge Electrical Designs (CED) Power 1401 data acquisition interface then recorded at a rate of 100 Hz on a second PC running the program Spike2 (see Fig. 2).

### Custom temperature Gauge

**Figure 3:**
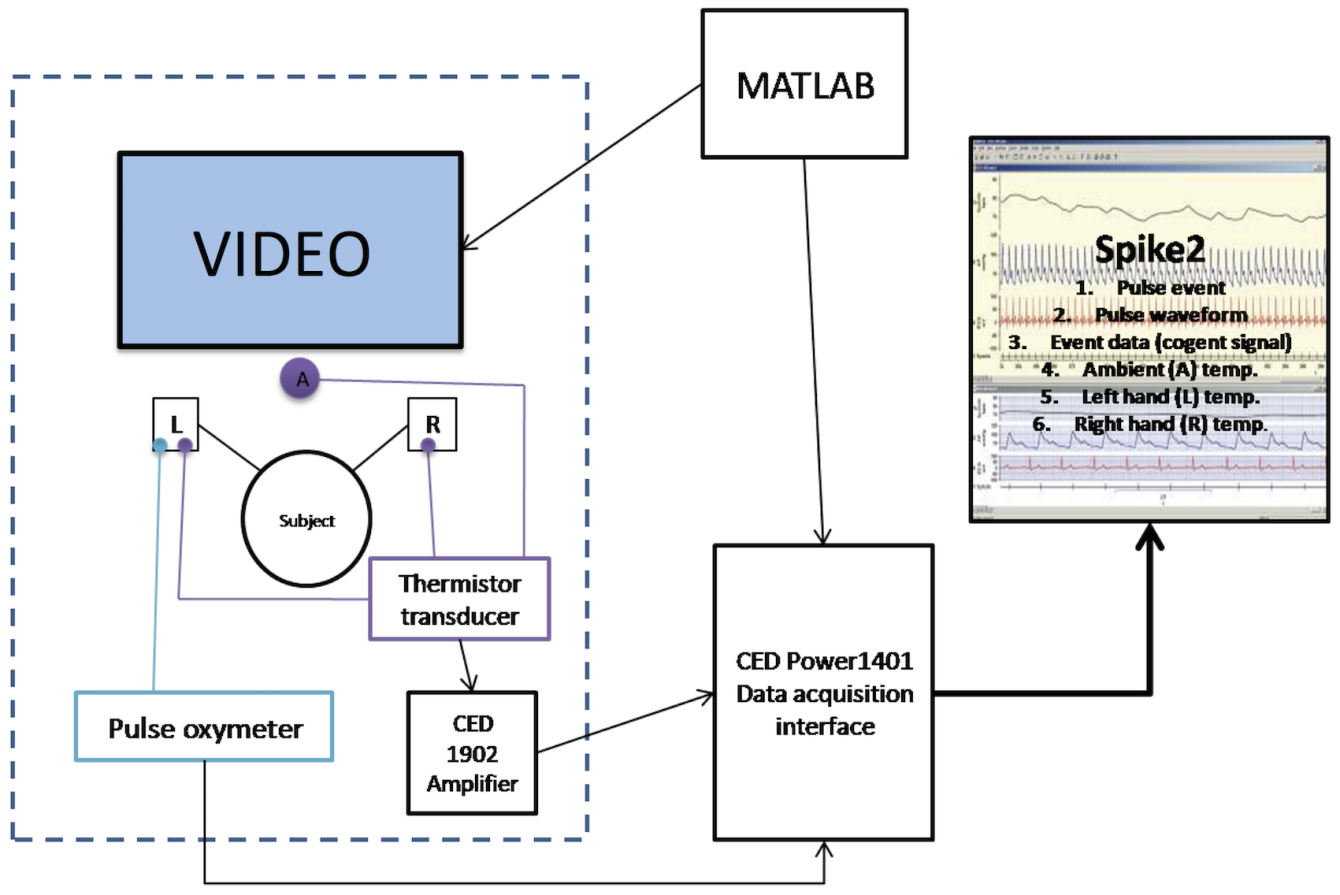
Temperature Gauge Characteristics. **A**) Graphical illustration of the structure of each of the three Wheatstone bridges each built using a 10 kΩ thermistor and three additional 10 kΩ thermistors. **B**) Resistance properties of the thermistors illustrating a near linear response over physiological temperature range of interest (20–40°C). **C**) Rate of change in resistance for the thermistors across the physiological temperature range. **D**) Theoretical temperature resolution of the temperature gauge across the physiological temperature range. **E**) Calibration of the temperature gauge.

Each 10 kΩ thermistor (EPCOS NTC B57861S103F40) was connected to a custom Wheatstone bridge built using three additional 10 kΩ (+/− 1%) resisters balanced with a rheostat (Fig. 3A). Each of the three Wheatstone bridges were then connected to a CED 1902 low noise, high-gain isolated pre-amplifier via 8-pin DIN plugs. Amplified potential differences were then passed to the CED 1401 data acquisition interface as described above. The thermistors used (Resistance (R_0_)  = 10 kΩ, B25/100 (beta)  = 3988K at a rated temperature (T_0_)  = 25°C) had a near linear resistance change (Equation 1, Fig. 3B) and rate of change of resistance (dR/dT) (Equation 2, Fig. 3C) over the physiological temperature range of interest (20–40°C).

The 10 Volt range and 16 bit resolution of the CED 1401 data acquisition devise coupled with the 30-fold gain of the CED 1902 amplifier enabled us to achieve a theoretical temperature sensitivity in the order of 0.00002°C that was near linear over the physiological temperature range of interest (20–40°C). (Equations 3–5, Fig. 3D). Finally, we calibrated the high-sensitivity temperature gauge against a digital thermometer (Kane-May 8004 digital thermometer) recording four points over the physiological temperature range 22–35°C. This demonstrated a linear relationship between voltage and temperature change (R2 = 0.98, p = 0.001, Temperature °C = 21.087× Voltage (V) +20.059 (Fig. 3E). To ensure that results were not influenced by potential differences in the sensitivity of the two finger thermistors half the participants had thermistor 1 attached to the left hand and thermistor 2 to the right hand and vice versa.

**Equation 1:** 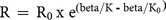


**Equation 2:** 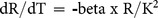


**Equation 3:** 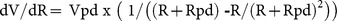


**Equation 4:** 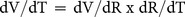


**Equation 5:** 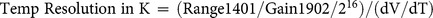


T =  temperature in °C, T_0_ = 25°C, R =  resistance, R_0_ =  10 kΩ, K (temperature in K)  = T+273.15, beta  = 3988 K, K_0_ = T_0_+273.15, Range1401  = 10 V, Gain1902  = 30, Rpd (potential divider resistance)  = 10,000, Vpd (potential divider supply voltage)  = 24 V.

### Physiological data analysis

Left and right hand temperature recordings were first corrected for minor fluctuations in room temperature by subtracting ambient temperature recordings at the equivalent time-point then de-trended to remove linear drifts across the experimental session. The container-filling phase (40 s), plus the subsequent 10 s (and equivalent pre-video playback period in the control condition) was used to measure video specific baseline temperature recordings. Video induced changes in participants' own hand temperature over the remaining 130 s were then calculated in 10 s epochs by subtracting baseline temperature from ambient corrected left and right hand temperatures. Data were then averaged within subjects to obtain a mean response to each video type e.g. warm/neutral/cool and left/right observed hand then analysed in second level repeated measures ANOVAs using SPSS 21. Video induced changes in heart rate were analysed in an equivalent manner.

### Empathy Questionnaires

Subjects completed two questionnaires: the Mehrabian Balanced Emotional Empathy Score (BEES) [36] and the Davis Interpersonal Reactivity Index (IRI) [37], [38]. The BEES contains 30 items e.g. “It upsets me to see someone being mistreated” rated on a 9-point agree/disagree scale and provides a well-validated measure of emotional empathy. The IRI contains 28 items rated on a 5-point does/does not describe me well scale. It provides a composite measure of dispositional empathy as well as sub-scales of Perspective Taking (PT) "I sometimes try to understand my friends better by imagining how things look from their perspective", Empathic Concern (EC) “I often have tender, concerned feelings for people less fortunate than me”, Personal Distress (PD) "Being in a tense emotional situation scares me” and Fantasy Scales (FS) "When I am reading an interesting story or novel, I imagine how I would feel if the events in the story were happening to me”.

BEES and Davis IRI total empathy score as well as the Davis sub-scores were then used in a step-wise multiple regression analysis in SPSS21 to investigate whether inter-individual differences in empathy predicted contagion of another's temperature change. Temperature contagion was defined as an individual's mean increase in temperature to all warm videos minus their mean decrease in temperature to all cool videos (averaged across both left and right hands).

## Results

### Ratings of observed temperature stimuli

**Figure 4:**
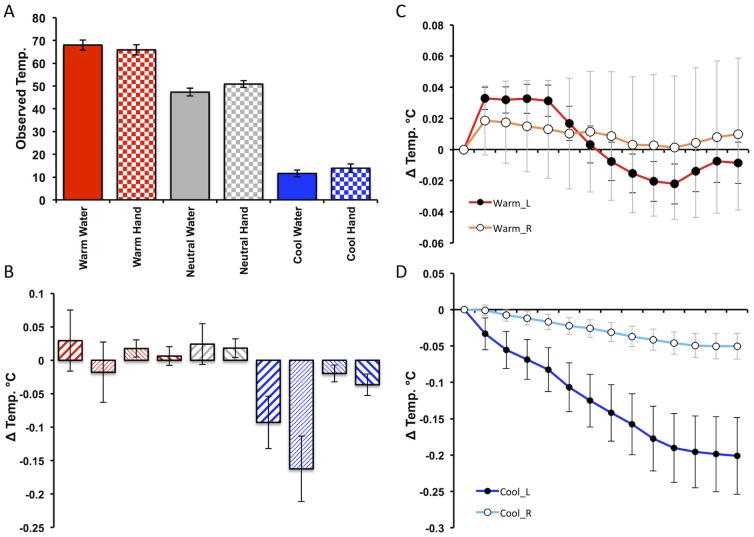
Behavioural and Temperature responses to Warm and Cool stimuli. **A**) Participants mean subjective ratings for the observed water temperature (“How Hot or Cold is the Water?”) and temperature of the actor's hand (“How Hot or Cold is the Actor's Hand?”) reported using a keyboard controlled visual analogue scale ranging from ‘Very Cold’ (far left) through ‘Neutral’ (centre) to ‘Very Hot’ (far right). **B**) Participants mean temperature change for their right (right leaning diagonals) and left (left leaning diagonals) when viewing warm (red) cold (blue) and neutral (grey) stimuli. Bold diagonals denote congruent responses (e.g. participants' left hand response when viewing left hand stimuli) and non-bold diagonals incongruent responses (e.g. e.g. participants' left hand response when viewing right hand stimuli). **C**) Mean time course response to viewing all warm videos for the left (red) and right (orange) hand displayed in 10 s epochs. **D**) Mean time course response to viewing all cold videos for the left (dark blue) and right (light blue) hand displayed in 10 s epochs.

Repeated measures ANOVAs with factors temperature (warm, cool) and observed hand (left, right) confirmed that our experimental manipulation significantly modulated participants' ratings of both the water and actors hand temperature, with both rated as appearing significantly warmer in the warm compared to cool conditions F_(1,34)_ = 449.25, p<0.001 and F_(1,34)_ = 256.67, p<0.001 respectively. There was no significant main effect of observed hand (left, right) or observed hand by temperature interaction for either rating demonstrating that the perceived temperature was equivalent for left and right hand video stimuli in both warm and cool conditions. We therefore collapsed ratings for observed (left, right) hand and repeated the ANOVAs including the neutral condition (warm, neutral, cool). This again confirmed that stimulus type (warm, neutral, cool) significantly affected ratings of the observed temperature for both the water and hand; F_(1,34)_ = 305.79, p<0.001 and F_(1,34)_ = 201.16, p<0.001 respectively. Post-hoc paired-sample t-tests confirmed significant differences (all p<0.001) between each pair of stimuli (Fig. 4A).

### Temperature analysis

Repeated measures ANOVA with factors observed temperature (warm, cool), observed hand (left, right) and participants own hand (left, right) demonstrated a significant main effect of observed temperature on participants' own hand temperature F_(1,35)_ = 13.83, p = 0.001, with post-hoc t-test demonstrating a significant reduction in participants' own left (t_(35)_ = −3.54, p = 0.001) and right (t_(35)_ = −2.33, p = 0.026) hand temperature during observation of cool videos but no change to warm videos (p>0.1) (Fig. 4B). We also observed a significant main effect of participant hand (F_(1,35)_ = 4.78, p = 0.036) and a significant participant hand × observed temperature interaction (F_(1,35)_ = 13.49, p = 0.001). Post-hoc t-tests showed that this was driven by a significantly greater reduction in left versus right hand temperature when viewing cool videos (paired t_(35)_ = −3.80, p = 0.001) demonstrating greater sensitivity of participants left hand to observed changes in temperature. Importantly, we did not observe significant observed × own hand or temperature × observed × own hand interactions (p>0.1) suggesting that induced changes in temperature were not influenced by laterality of the observed hand. Finally, there was no change in participants' own left or right hand temperature when they observed the neutral videos (p>0.1) (data illustrated in Fig. 4B).

The time scale of induced changes in participants' own hand temperature when viewing warm and videos are illustrated in Fig. 4C and 4D respectively and demonstrate a maximum 0.2°C temperature drop 2 minutes into the cool videos and a maximum 0.033°C temperature rise occurring 10–50 s after onset of the warm videos.

### Heart rate analysis

Repeated measures ANOVA with factors observed temperature (warm, cool), observed hand (left, right) and participants own hand (left, right) demonstrated no significant effect of observed temperature on participants own heart rate (all main effects and interactions p>0.1).

### Relationship between temperature contagion and empathy

**Figure 5:**
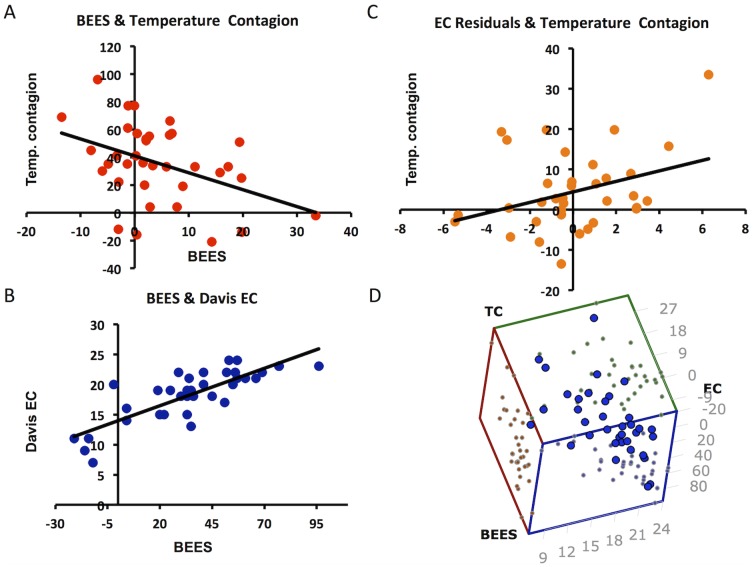
Relationship between sensitivity to temperature contagion, BEES and the empathic concern subscale of the Davis IRI. A) Relationship between sensitivity to temperature contagion and BEES. B) Relationship between BEES and empathic concern (EC) subscale of the Davis IRI. C) Relationship between EC residuals (after regressing out relationship with BEES) and sensitivity to temperature contagion. D) 3D scatter plot illustrating the relationship between sensitivity to temperature contagion (TC), BEES and empathic concern subscale of ARI (EC).

Finally we investigated whether self-reported empathy scores predicted an individual's contagion of another's observed temperature. Multiple regression analysis demonstrated that both the BEES and the Empathic Concern (EC) sub-scale of the Davis IRI (but not the other Davis sub-scales) significantly predicted contagion of observed temperature changes (F_(2,35)_ = 6.82, p<0.003) with an adjusted R^2^ = 0.25. Exploration of the factors within this model demonstrated a nuanced relationship between empathy and contagion of another's temperature change; specifically in this model the BEES (which provides a single composite measures of empathy) negatively predicted temperature contagion (t_(35)_ = −3.68, p = 0.001, β = −0.92) while the EC sub-scale of the Davis (which selectively measures empathic concern) positively predicted temperature contagion (t_(35)_ = 2.79, p = 0.009, β = 0.69) (Fig. 5).

## Discussion

Here we aimed to investigate emotional contagion in the domain of body temperature by showing healthy volunteers video clips of actors with their hands in warm or cold water while simultaneously recording their own right and left hand temperature. We show that healthy participants exhibit contagion of another's hand temperature even in the absence of visible emotional or facial cues. This temperature contagion was particularly prominent for cool stimuli and was more marked for the observers' left hand. However, it should be noted that ice cubes were clearly visible throughout the cool condition but steam visible only at the beginning of the warm videos which may have contributed to this finding. We found no interaction between the laterality of the hand observed and experiencing the change in temperature arguing against common rotational or mirror symmetry effects described in naturalistic social interactions [7]; though note that viewed hands were oriented perpendicular to their own. Interestingly, self-report measures of empathy additionally predicted inter-individual differences in sensitivity to temperature contagion. This demonstration of contagion of observed body temperature extends the range of human mimetic responses to another axis of the autonomic nervous system. It also lends empirical support to extension of perception-action mechanisms to non-volitional, non-emotional responses exclusively mediated by the autonomic nervous system [9], [10].

Maintaining a stable internal thermal environment is critical to the life-preserving actions of bioactive proteins, such as enzymes and ion channels. As a consequence, core body temperature is rigidly regulated by the brains of all homeothermic animals including humans through a variety of involuntary thermoregulatory responses, such as shivering and non-shivering thermogenesis, cutaneous vasomotor responses, sweating, piloerection and panting [32]. In response to a reduction in environmental temperature skin temperature falls rapidly and triggers firing of strategically located COOL-sensitive neurons [39]. Feed-forward thermal afferent information is then relayed via the external lateral parabrachial nucleus (LPBel) to the hypothalamic thermoregulatory centre in the preoptic area (POA) resulting in an inhibition of its tonic discharge. This ultimately results in disinhibition of sympathetic premotor neurons within the rostral medullary raphe (rMR) orchestrating cutaneous vasoconstriction, tachycardia, skeletal muscle shivering [32], [40] and non-shivering thermogenesis in brown adipose tissue [12].

Interestingly, different effectors mechanisms are associated with partially separable central control systems [41], expressed physiologically as a greater sensitivity of vasoconstrictive responses to temperature change [33]. This difference in central control mechanism may also underpin why, in our current study, we saw isolated changes in hand temperature (likely mediated by a direct POA-rMR pathway) but not heart rate (mediated by an intermediate projection to the dorsomedial hypothalamus (DMH)) [32]. WARM sensitive neurons projecting via the dorsal parabrachial nucleus (LPBd) play a similar role in orchestrating cutaneous vasodilation and tachycardia in response to environmental warming [42].

In addition to bottom-up feed-forward pathways triggered by changes in skin temperature, thermoregulatory responses are also sensitive to top-down influences for example though visual imagery, temperature biofeedback and hypnotic suggestion [43]. This was first highlighted by Hadfield in 1920 in a case report of a patient who was able to selectively increase and decrease their right and left hand temperature by almost 3°C through suggestions of heat or cold [44]. Subsequently, similar selective increases and decreases in left and right hand temperature have been demonstrated in response to hypnotic suggestion in adults [45] and children [46] as well as biofeedback [47], [48], instructed imagery [49] or combinations of these techniques [50], [51]. More recently, synchronous changes in facial temperature have also been reported in mothers observing their child at play [52]. A review of these studies [43] has highlighted that temperature decreases are typically easier to elicit and of greater magnitude than temperature increases, and occur in the absence of heart rate change, as we observed. These top-down influences on thermoregulatory responses have also been exploited clinically in the treatment of Raynauld's syndrome [53], [54], though large inter-individual variability in ability to regulate finger temperature has limited its more widespread clinical adoption [55].

Direct cooling of the hand has been shown to increase blood flow (an indirect measure of neuronal activity) within the posterior insula [56], a region proposed to provide a cortical representation of all visceral afferent input [57], [58]. Whether similar increases in insula activity are also associated with temperature changes observed in a conspecific is currently unknown, though the wealth of fMRI studies showing shared empathic in other domains would predict that they would. In this regard, it is also instructive to note that in monkeys many POA thermosensitive neurons are additionally affected by non-thermal emotional stimuli such as rewards or aversive stimuli [59] suggesting that hypothalamic POA-rMR effector pathways may be recruited by top-down cognitive processes.

Insight into the mechanism underlying temperature contagion may also be usefully informed by studies of disrupted body ownership induced either experimentally using the rubber hand illusion [35] or in the clinical disorder cold-type complex regional pain syndrome (CRPS) [60]. In both of these conditions unilateral disruption of body ownership is associated with a localised reduction in body temperature suggesting that the conscious sense of our physical self and its physiological regulation are linked. During experimental induction of the rubber hand illusion activity changes are observed within insula cortex as well as premotor and intraparietal cortex [61] suggesting a potential role for the insula in reported temperature changes. In CRPS patients (a neurological disorder associated with pain, abnormal temperature regulation and often dystonia in a single limb) changes in limb temperature were reported dependent upon its location in space [60]. For example, when the affected (cool) limb was moved across the midline its temperature spontaneously increased with a converse effect described for the healthy limb. On the basis of these findings the authors argued for a space-based rather than somatotopic frame of reference with descending projections from parietal cortex onto brainstem autonomic centres hypothesised as the mechanism through which changes in the spatial location of the limb result in associated temperature change. It is thus possible that inter-personal comparator processes within the intraparietal junction play a similar role in temperature contagion.

Finally, in contrast to our prediction of a simple relationship between sensitivity to temperature contagion and empathy score we found a more nuanced relationship. Specifically, an inverse relationship between BEES emotional empathy score and sensitivity to temperature contagion. i.e. those individual who scored highest on the BEES showed the least sensitivity to temperature contagion. However, when we included both the BEES and the Davis IRI (including subscores) into a stepwise linear regression analysis we showed that though BEES continued to show a negative relationship to temperature contagion the empathic concern subscale of the IRI showed a positive relationship. The basis for these findings is currently unclear, though may relate to subtle differences in the concepts captured by the BEES and EC scales. For example, though we showed a tight positive correlation between BEES and EC scores (R^2^ = 0.65, p<0.001) across participants it was participants with relatively high EC compared to BEES scores that showed the greatest propensity to temperature contagion. Alternately this finding may relate to the nature of our experimental stimuli in which we were careful not to show discernable emotional cues. It would be important for future studies to clarify the precise nature of the relationship between individual differences in emotion contagion, empathic distress and concern particularly to such low-level contagion phenomena.

To conclude, here we show that healthy individuals are sensitive to observable signals of another's peripheral body temperature and further show contagion of their temperature, particularly in the context of cold. Inter-individual differences in temperature contagion are marked and show a complex relationship to inter-individual difference in empathy. Interestingly, abnormal temperature regulation is also observed in disorders of social cognition such as autism [62] and Schizophenia [63] suggesting interest in measuring temperature contagion in these populations.
